# The Temporal Requirements for Insulin Signaling During Development in *Drosophila*

**DOI:** 10.1371/journal.pbio.0030289

**Published:** 2005-08-16

**Authors:** Alexander W Shingleton, Jayatri Das, Lucio Vinicius, David L Stern

**Affiliations:** **1**Ecology and Evolutionary Biology, Princeton University, Princeton, New Jersey, United States of America,; **2**Leverhulme Centre for Human Evolutionary Studies, University of Cambridge, Cambridge, United Kingdom; Columbia UniversityUnited States of America

## Abstract

Recent studies have indicated that the insulin-signaling pathway controls body and organ size in *Drosophila*, and most metazoans, by signaling nutritional conditions to the growing organs. The temporal requirements for insulin signaling during development are, however, unknown. Using a temperature-sensitive insulin receptor (*Inr*) mutation in *Drosophila,* we show that the developmental requirements for Inr activity are organ specific and vary in time. Early in development, before larvae reach the “critical size” (the size at which they commit to metamorphosis and can complete development without further feeding), Inr activity influences total development time but not final body and organ size. After critical size, Inr activity no longer affects total development time but does influence final body and organ size. Final body size is affected by Inr activity from critical size until pupariation, whereas final organ size is sensitive to Inr activity from critical size until early pupal development. In addition, different organs show different sensitivities to changes in Inr activity for different periods of development, implicating the insulin pathway in the control of organ allometry. The reduction in Inr activity is accompanied by a two-fold increase in free-sugar levels, similar to the effect of reduced insulin signaling in mammals. Finally, we find that varying the magnitude of Inr activity has different effects on cell size and cell number in the fly wing, providing a potential linkage between the mode of action of insulin signaling and the distinct downstream controls of cell size and number. We present a model that incorporates the effects of the insulin-signaling pathway into the *Drosophila* life cycle. We hypothesize that the insulin-signaling pathway controls such diverse effects as total developmental time, total body size and organ size through its effects on the rate of cell growth, and proliferation in different organs.

## Abstract 2

Studies using a temperature-sensitive insulin receptor elucidate the temporal requirements for insulin signaling in Drosophila; insulin signaling at different times during development affects many characters, such as total developmental time, total body size and organ size.

## Introduction

Development in multicellular animals is a process that involves both tight control and flexibility in the regulation of cell size and cell number. Tight control is necessary to produce an animal in which each organ is of an appropriate size relative to the whole body. Flexibility is necessary to produce an animal in which the whole body is of an appropriate size relative to environmental conditions. One key environmental factor shown to influence size is nutrition. In animals as diverse as humans and flies, malnutrition delays development and reduces adult body and organ size. Recent studies indicate that the insulin-signaling pathway (Gene Ontology ID GO:0008286) coordinates growth with nutritional condition in most metazoans, and is remarkably conserved. In *Drosophila,* expression of insulin-like peptides is nutritionally regulated [1], as is expression of IGF1 (insulin-like growth factor 1) in developing rats [2]. Flies carrying mutations of the insulin receptor *(Inr)* and mice carrying mutations of the IGF1 receptor show delayed development and growth deficiency with a reduction in body and organ size [3–6]. These and similar “knock out” experiments have demonstrated the gross effects of the insulin-signaling pathway on adult phenotype. Nevertheless, little is known of how the pathway acts during development to affect changes in the adult. In mice, such elucidation is hampered by the inaccessibility of the developing fetus enclosed in the uterus. In flies, however, developing larvae can be easily studied and manipulated.

Here we explore the temporal requirement for insulin signaling in developing *Drosophila* *melanogaster.* In *Drosophila,* development proceeds through three larval instars to pupariation, pupation, and finally adult eclosion. Feeding is restricted to the first (L1), second (L2), and most of the third (L3) instar. Early in the third instar, the larvae reach a “critical size” at which point they have acquired sufficient nutrients to complete development in the absence of food [7–10]. After critical size is attained, larvae stop feeding, leave the food, and search for a pupariation site. There is, however, a delay between the time critical size is reached and the time larvae stop feeding and reach their maximum body size. Both critical size and the time between reaching critical size and the cessation of feeding are unaffected by nutrition [8,10]. Consequently, nutrition ostensibly affects final adult size through the amount of feeding in the fixed period between critical size and pupariation [10].

Because feeding affects final size only in the period between critical size and pupariation [8,10], one may predict that insulin signaling affects size only during the same period. However, organ growth continues well after pupariation [11,12] and is autonomously modulated by the insulin-signaling pathway [5,13]. Insulin signaling may therefore continue to influence size after pupariation. Details of the temporal requirements for insulin signaling are unknown in *Drosophila,* but a recent study on the *Drosophila* forkhead transcription factor (dFOXO) suggests that they vary during development [14]. Unphosphorylated dFOXO negatively regulates growth, but activation of the insulin receptor signaling pathway induces dFOXO phosphorylation, excluding it from the nucleus and inhibiting its activity [15,16]. Constitutive activation of dFOXO in the first and second instars causes developmental arrest, whereas dFOXO activation in the third instar causes a reduction in adult size [14]. Work on *Caenorhabditis elegans* also indicates that the timing requirements for insulin signaling might vary during the lifecycle. In *C. elegans*, the pathway acts during early development to regulate diapause, and during adulthood to influence ageing [17]. Resolving the links between larval size, nutrition, and developmental progression on the one hand, and the time-dependence of insulin-pathway gene effects on the other, requires a more precise understanding of the temporal requirements for insulin signaling during development.

We have used a temperature-sensitive mutation of the insulin receptor to investigate the role of the insulin-signaling pathway during different stages of development in *Drosophila*. By reducing insulin receptor activity at different points in development, we identify the periods of development during which insulin signaling affects adult phenotype. We show that total developmental time is affected by changes in insulin signaling only if those changes occur early in development, before a larva has reached critical size. In contrast, final body and organ size are affected by changes in insulin signaling only if those changes occur late in development, once a larva has passed critical size. Insulin signaling continues to influence organ size, but not body size, after larvae have stopped feeding and throughout much of pupal development. Not all organs respond equally to changes in insulin signaling, however. We find that the genitals show a limited response to suppression of insulin signaling in *Inr^E19^/Inr^GC25^* flies, implicating insulin signaling in the control of allometries.

## Results

### Temperature-Sensitive Suppression of the Insulin Receptor

**Figure 1:**
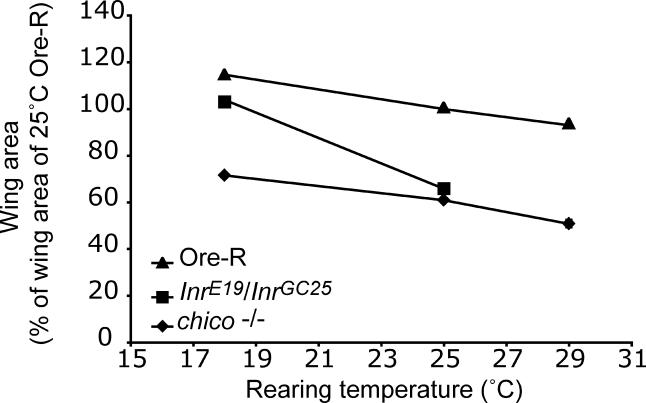
Temperature Sensitivity in *Inr^E19^/Inr^GC25^* Flies Increasing the rearing temperature of *Inr* ^E19^/*Inr^GC25^* females from 18 °C to 25 °C causes a reduction in wing area from approximately wild-type (Oregon-R [Ore-R]) to that of an insulin pathway mutant *(chico)*. Wing size is expressed as percentage area of Oregon-R female wing at 25 °C. No *Inr^E19^/Inr^GC25^* flies survived rearing at 29 °C. The standard errors are smaller than the markers.

During a study of the interaction of environmental factors and the insulin-signaling pathway, we discovered that flies trans-heteroallelic for insulin receptor mutations *Inr^E19^* and *Inr^GC25^* show a temperature-sensitive suppression of the insulin pathway. We initially reared *Inr^E19^/Inr^GC25^* flies, wild-type Oregon-R flies, and flies homozygous for *chico^1^*, at 18 °C, 25 °C, and 29 °C, and measured the wing areas of the adults. Chico is the fly ortholog of the insulin receptor substrate. *chico^1^* is a null mutation, and homozygotes show a partial loss of insulin receptor function with a more than 50% reduction in body size relative to wild-type and a much reduced viability [5]. Both Oregon-R and *chico^1^* homozygote flies showed a smaller wing area with higher rearing temperature (Figure 1). The response to temperature is the same in both genotypes (the lines relating wing size to temperature are parallel in Figure 1), indicating that temperature influences size independent of the insulin-signaling pathway. However, *Inr^E19^/Inr^GC25^* flies have a wing size similar to Oregon-R flies when reared at 18 °C but a wing size similar to *chico^1^* mutants when reared at 25 °C (Figure 1). This suggests that *Inr^E19^/Inr^GC25^* flies show temperature-sensitive suppression of Inr activity. Changing the rearing temperature does not, however, simply switch Inr “on” or “off” in *Inr^E19^/Inr^GC25^* flies. At 18 °C, *Inr^E19^/Inr^GC25^* flies have smaller wings than wild-type flies, suggesting that the insulin receptor is still partially suppressed at this temperature. Additionally, very few *Inr^E19^/Inr^GC25^* flies survived to adulthood when raised at 25 °C. and none survived at 29 °C. The difference between *Inr^E19^/Inr^GC25^* flies reared at 18 °C and 25 °C appears, therefore, to be a consequence of the degree to which Inr is suppressed.

**Figure 2:**
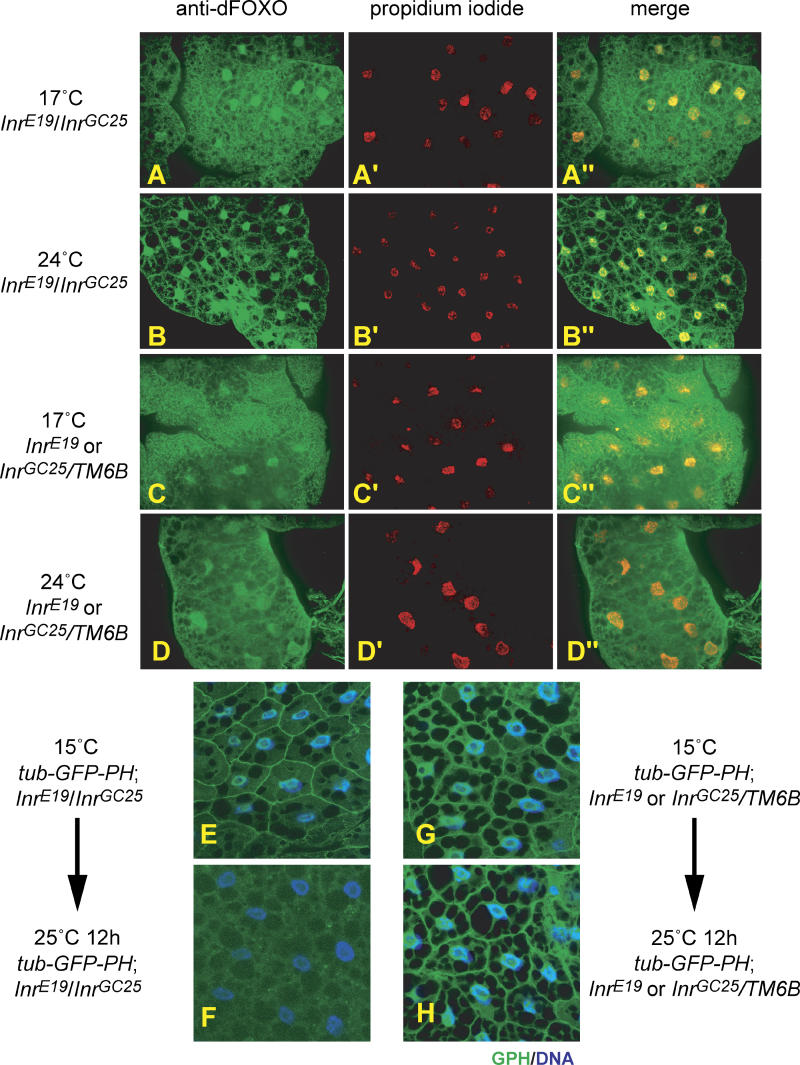
Increasing the Temperature of *Inr^E19^/Inr^GC25^* Flies Suppresses the Insulin-Signaling Pathway The dFOXO panel shows localization of dFOXO protein in the fat body, the propidium iodide panel shows the position of the nuclei, and the merge panel clarifies the degree of dFOXO localization to the nuclei. (A) Endogenous dFOXO in the fat body of *Inr^E19^/Inrv^GC25^* third instar larvae has weak nuclear localization at 17 °C. (B) Increase in rearing temperature causes a decrease in cytoplasmic distribution and an increase in nuclear localization of dFOXO, consistent with a decrease in the level of insulin signaling (C and D) Temperature has no detectable effect on dFOXO localization in *Inr^E19^/TM3* control flies. (E) GPH membrane localization reveals high levels of insulin signaling in the fat body of *Inr^E19^/Inr^GC25^* second instar larvae reared at 15 °C. GPH is in green, DNA is stained blue. (F) This localization is lost when the larvae are moved to 25 °C for 12 h, consistent with a decrease in the level of insulin signaling. (G and H) Temperature has no detectable effect on GPH membrane localization in *Inr^E19^*/TM3 control flies.

To confirm that these temperature-dependent effects were due to changes in Inr activity, we assessed the activity of the insulin-signaling pathway in *Inr^E19^/Inr^GC25^* flies reared at different temperatures. First, we looked at the cellular localization of dFOXO in *Inr^E19^/Inr^GC25^* late third instar larvae reared at 17 °C and at 24 °C. Down-regulation of the insulin-signaling pathway dephosphorylates dFOXO and causes it to move from the cytoplasm to the nucleus. Antibody stains with anti-dFOXO show an increase in nuclear versus cytoplasmic localization of dFOXO in *Inr^E19^/Inr^GC25^* larvae when reared at 24 °C relative to 17 °C (Figure 2A and 2B). We did not observe change in nuclear versus cytoplasmic staining for *Inr^E19^* or *Inr^GC25^/TM6B-Tb* controls reared at 17 °C versus 25 °C (Figure 2C and 2D). Second, we assessed Inr activity in *Inr^E19^/Inr^GC25^* second instar larvae using the *tGPH* reporter gene [18]. *tGPH* is under the control of the *Drosophila* ß-tubulin promotor and produces a GFP (green fluorescent protein) fused to the pleckstrin homology domain of the receptor for phosphoinositides-1. Under the action of phosophoinositide 3-kinase (PI3K), this fusion protein, GPH, becomes localized to the cell membrane. PI3K is itself activated by the insulin receptor, and so increased localization of GPH to the cell membrane indicates increased Inr activity. In *Inr^E19^/Inr^GC25^* larvae reared at 15 °C, GPH is localized to the membrane, but this localization is lost when the larvae are moved to 25 °C for 12 h (Figure 2E and 2F). Localization does not appear to be lost at 25 °C in *Inr^E19^* or *Inr^GC25^/TM6B-Tb* controls (Figure 2G and 2H). The phenotypic effects of temperature on *Inr^E19^/Inr^GC25^* flies do, therefore, appear to be a consequence of temperature-sensitive suppression of the insulin-signaling pathway. However, we do not know the molecular basis for the temperature sensitivity of the *Inr* ^E19^ */Inr* ^GC25^ transheterozygotes. It may arise from temperature-sensitive expression of one or both of the mutant alleles or from decreased function of the mutant receptor at higher temperatures.

Due to the low viability of *Inr^E19^/Inr^GC25^* flies reared at 25 °C, all subsequent experiments involved comparisons of flies reared at 17 °C with flies reared at 24 °C. Further, we used the *Inr^E19^/TM3* siblings reared under identical conditions as controls. *Inr^E19^/TM3* flies show only a moderate reduction in Inr activity and have a slight reduction in body size relative to wild-type [4]. Because temperature affects overall body-size in wild-type flies [19], it is necessary to distinguish between changes in *Inr^E19^/Inr^GC25^* phenotype that are a consequence of changes in the level of *Inr* expression from those resulting from changes in rearing temperature. To do this we report the phenotype of *Inr^E19^/Inr^GC25^* flies as a percentage of the phenotype of *Inr^E19^/TM3* control flies that have undergone temperature shifts at the same developmental time.

### The Changing Role of Insulin Signaling during Development

We used the temperature-sensitive *Inr* mutants to investigate the role of the insulin pathway during *Drosophila* development. We reduced Inr activity during different periods of development by transferring *Inr^E19^/Inr^GC25^* flies from a permissive 17 °C to a restrictive 24 °C at different points in development. After the switch, the flies were left to complete development at 24 °C. We were able to identify temperature-sensitive periods (TSPs) during which increasingly earlier switches from 17 °C to 24 °C resulted in increasingly abnormal phenotypes.

**Figure 3:**
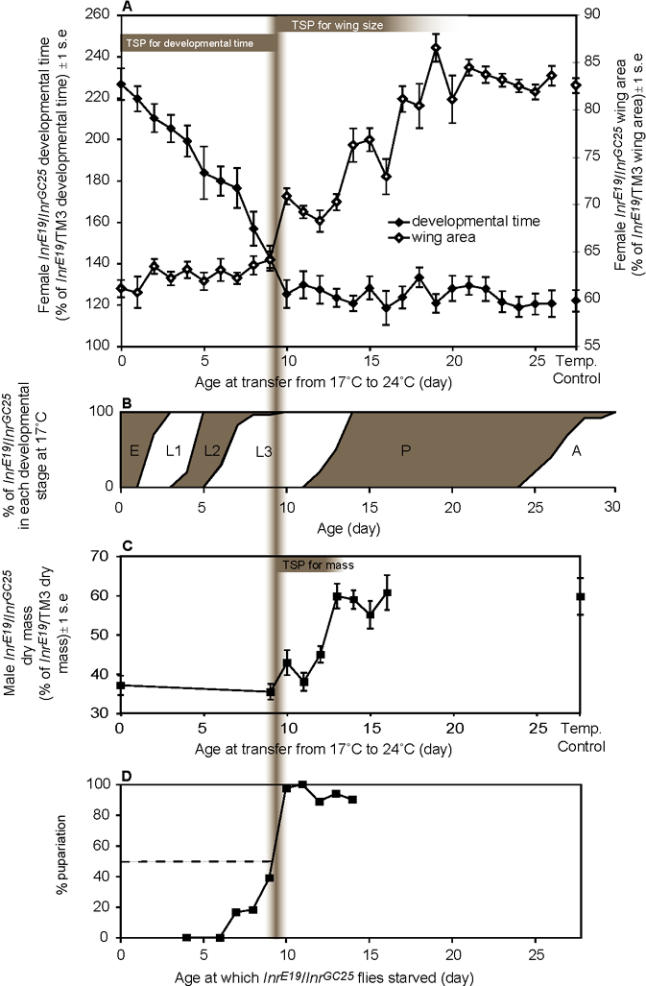
Suppression of *Inr* Expression in *Inr^E19^/Inr^GC25^* Flies Affects Developmental Time and Adult Size (A) Developmental time and adult wing size of *Inr^E19^/Inr^GC25^* females switched from 17 °C to 24 °C increasingly late in development, expressed as percentage of developmental time and adult wing size of *Inr^E19^/TM3* females maintained under identical thermal conditions. Temperature-control flies were maintained at 17 °C throughout development. TSPs of female *Inr^E19^/Inr^GC25^* for wing area and delayed eclosion can be seen as regions of the chart where switching from 17 °C to 24 °C increasingly early in development results in increasingly abnormal phenotypes (that is, where the gradient of the relationship between switch day and phenotype is non-zero). For delayed adult eclosion, the TSP of female *Inr^E19^/Inr^GC25^* is before the ninth day of development at 17 °C. For reduced wing size, the TSP of female *Inr^E19^/Inr^GC25^* is between the ninth and approximately the 20th day of development at 17 °C. (B) The stages of development of *Inr^E19^/Inr^GC25^* flies at 17 °C (A, adult; E, embryo; L1, first instar; L2, second instar; L3, third instar; P, pupae). The point at which suppression of the insulin pathway changes from delaying adult development to reducing adult wing size occurs approximately 40% into the third instar (vertical gray bar) (C) Dry mass of *Inr^E19^/Inr^GC25^* males switched from 17 °C to 24 °C at different points in development, expressed as percentage of dry mass of *Inr^InrE19^/TM3* males maintained under identical thermal conditions. The TSP of male *Inr^E19^/Inr^GC25^* for reduced adult mass is after the ninth day of development but before pupariation. (D) Proportion of 17 °C *Inr^E19^/Inr^GC25^* larvae pupariating when completely starved at different points in development. The point at which 50% of larvae pupariate in the absence of food marks the critical size. The critical size is reached approximately 40% through the third instar and coincides with the end of the TSP for delayed eclosion and the beginning of the TSP for reduced wing size and adult dry mass (vertical gray bar). All pupariating larvae successfully completed metamorphosis and eclosed as adults.

**Figure 4:**
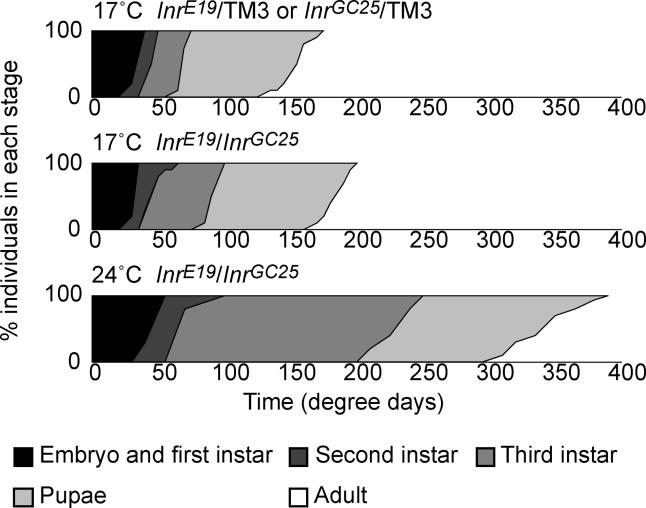
Developmental Delay in 24 °C *Inr^E19^/Inr^GC25^* Flies Occurs primarily through Elongation of the Third Larval Instar Area shows percentage of individuals (*n* = 20) in each developmental stage at different times in development. Time is shown in DDs to control for the effect of temperature on developmental rate.

Total developmental time is sensitive to Inr activity only before the middle of the third larval instar (Figure 3A). Switching *Inr^E19^/Inr^GC25^* flies from 17 °C to 24 °C changes the time to adult eclosion during the first 9 d of development; the earlier the switch, the greater the delay in eclosion. After the ninth day of development at 17 °C, when the flies are approximately 40% through the third instar (Figure 3B), a shift to the restrictive temperature does not delay adult eclosion. At 17 °C, *Inr^E19^/Inr^GC25^* flies show a slight delay in eclosion relative to *Inr^E19^/TM3* flies, suggesting that Inr activity is still a little impaired at this temperature. The delay in *Inr^E19^/Inr^GC25^* flies reared at both 17 °C and 24 °C relative to the *Inr^E19^/TM3* controls occurs predominantly through extension of the third instar (Figure 4).

In contrast, adult wing area is sensitive to Inr activity only during late third instar and early pupation (see Figure 3A). Switching *Inr^E19^/Inr^GC25^* flies from 17 °C to 24 °C changes adult wing size between day 9 and 20; the earlier the shift, the smaller the wings The precise TSP of female *Inr^E19^/Inr^GC25^* flies for wing area may be smaller than implied by Figure 3A because the data are based on population rather than individual measures. The wings may be insensitive to changes in Inr activity as early as day 17. Before day 9, however, shifting the flies to the restrictive temperature earlier in development has no additional effect on adult wing size. At 17 °C, the wings of *Inr^E19^/Inr^GC25^* flies are slightly smaller than in *Inr^E19^/TM3* control flies, again suggesting that Inr activity is marginally reduced at this temperature.

We tested whether total body mass was sensitive to Inr activity over the same period as wing size. We compared the dry mass of adult *Inr^E19^/Inr^GC25^* males reared under several thermal conditions: 24 °C, 17 °C, and a series of samples switched from 17 °C to 24 °C on days 9 through 16 (pupariation is at approximately day 13 at 17 °C). Adult body mass is sensitive to reduction in Inr activity between day 9 and day 13 at 17 °C (see Figure 3C). Shifting *Inr^E19^/Inr^GC25^* flies to the restrictive temperature after pupariation, and therefore after larvae have stopped feeding, has no influence on adult mass. Shifting the flies earlier than day 9 has no additional effect on adult body mass.

Inr activity therefore influences total development time, adult wing size, and adult mass for different periods of development. We tested whether the time at which down-regulation of the insulin pathway switches from delaying development to reducing the size of the resulting flies coincides with attainment of critical size. In practice, the critical size is determined as the size at which 50% of larvae proceed to pupariation in the absence of food [8]. We measured the timing of critical size in *Inr^E19^/Inr^GC25^* flies reared at 17 °C under our experimental conditions and found that it is attained at approximately day 9 (see Figure 3D), coinciding with the time at which Inr activity switches from affecting developmental time to body size. Therefore, larval critical size coincides with the shift in Inr function.

### A Reduction in Inr Activity Changes Body Chemistry

To better understand why reduction of Inr activity after pupariation influenced adult wing size but not overall body mass, we measured the protein, lipid, glycogen, and sugar content of *Inr^E19^/Inr^GC25^* flies reared at 17 °C up to pupariation, then either switched to 24 °C or maintained at 17 °C.

Reducing Inr activity after pupariation results in an approximate doubling of free-sugar concentration in *Inr^E19^/Inr^GC25^* flies (Table 1). There is no similar response in protein, glycogen, or lipid levels. Lipid levels are, however, elevated in both *Inr^E19^/Inr^GC25^* flies maintained at 17 °C and those switched to 24 °C at pupariation relative to *Inr^E19^/TM3* flies (Table 1). *Inr^E19^/Inr^GC25^* flies grown at a restrictive 24 °C for their entire development also have elevated lipid levels (138% ± 13.3 of *Inr^E19^/TM3* flies). This suggests that the slight deficiency in insulin signaling at 17 °C may account for the entire effect on lipid levels.

**Table 1:**
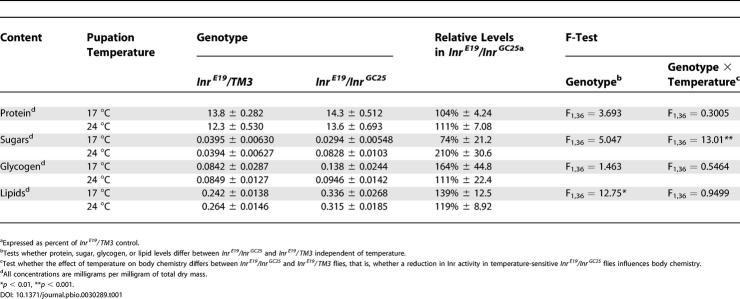
Protein, Sugar, Glycogen, and Lipid Content of *Inr^E19^/Inr^GC25^* and *Inr^E19^/TM3* Flies Reared at 17 °C until Pupariation and then Switched to 24 °C or Maintained at 17 °C

### A Reduction in Inr Activity Affects Different Organs Differently

Different organs typically grow at different rates in developing animals, a phenomenon called “allometry.” Patterns of allometry in populations can also result from organs growing at the same rate but starting and stopping growth at different times in development [20]. We investigated whether the insulin-signaling pathway might be involved in the regulation of allometry by examining whether different organs respond differently to reductions in Inr activity induced by a temperature shift of *Inr^E19^/Inr^GC25^* flies from 17 °C to 24 °C. We examined organs located at the anterior, median, and posterior of males: the maxillary palp, wing, and genital arch. We chose the maxillary palp rather than another anterior organ because it is similar in size to the genital arch, allowing us to control for absolute size in this comparison.

**Figure 5:**
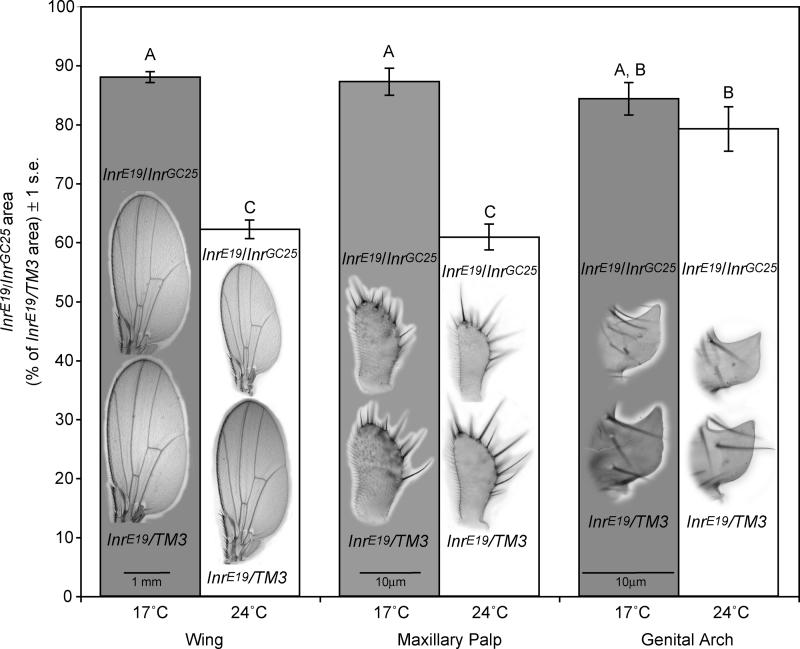
Different Organs Respond Differently to Suppression of *Inr* Activity Bars show organ area in *Inr^E19^/Inr^GC25^* males as a percentage of area in *Inr^E19^/TM3* males, to control for temperature effects. Bars with different letters indicate organs that differ: A, B, and C are significantly different at α = 0.05 (Tukey-Kramer pairwise comparison). Mean areas of all organs given in Table S1. s.e., standard error.

Figure 5 shows the relative areas of wings, genital arch, and maxillary palps of male *Inr^E19^/Inr^GC25^* and male *Inr^E19^/TM3* flies reared at either 17 °C or 24 °C. At 17 °C, all three organs are smaller in *Inr^E19^/Inr^GC25^* males than *Inr^E19^/TM3* males. At 24 °C, the wing and maxillary palps are further reduced in *Inr^E19^/Inr^GC25^* males, consistent with a further reduction in the level of Inr activity. In contrast, there is no difference in the size of the genital arches, relative to *Inr^E19^/TM3* males, in flies reared at 17 °C and 24 °C.

This suggests that the size of the genital arches may not be regulated by Inr activity. However, the arches are smaller in *Inr^E19^/Inr^GC25^* males than in *Inr^E19^/TM3* males at both rearing temperatures. It is possible that this size difference is a consequence of genetic background, unrelated to differences in Inr activity in the two genotypes. Alternatively, the genital arches may show a limited response to changes in Inr activity. They may be sensitive to a mild reduction in Inr activity experienced by *Inr^E19^/Inr^GC25^* flies reared at 17 °C, but be insensitive to a further reduction experienced by *Inr^E19^/Inr^GC25^* flies reared at 24 °C.

To distinguish between these two hypotheses, we generated large Minute clones homozygous for *chico^1^*. Homozygous *chico^1^* clones produce phenotypes identical to mutant *Inr* clones [13] and autonomously cause a dramatic size reduction in fly wings and eyes, whereas the heterozygous *chico^1^* cells behave as wild-type [5]. We identified males that were homozygous for *chico^1^* throughout one side of the genitals and heterozygous for *chico^1^* on the other. If genital size is independent of the insulin-signaling pathway, then the genitals from either compartment should be identical in size. Each comparison was made within a single male, automatically controlling for total body size. We found that genital arches consisting of mutant *chico^1^* clones were 16% smaller than paired genital arches on the same male (genital arch area: *chico^1^* mutant = 2,840 ± 60 μm^2^, *chico^1^* wild-type = 3,230 ± 110 μm^2^, paired-sample *t* test: *n* = 6, *t* = 1.67, *p* = 0.0214). This reduction of 16% is consistent with the 16% reduction observed in *Inr^E19^/Inr^GC25^* males relative to controls (Figure 5). In contrast, maxillary palps consisting of mutant *chico^1^* clones were 45% smaller than paired palps on the same male (maxillary palp area: *chico^1^* mutant = 4,820 ± 100 μm^2^, *chico* wild-type = 8,640 ± 170 μm^2^, *n* = 7). The genital arches do, therefore, show a limited response to changes in insulin signaling. They are sensitive to a mild reduction in Inr activity, such as observed in *Inr^E19^/Inr^GC25^* flies reared at 17 °C. Further reduction in Inr activity, such as observed in *Inr^E19^/Inr^GC25^* flies reared at 24 °C, has no additional effect on genital arch size but does have an effect on maxillary palp and wing size.

### A Reduction in Inr Activity Affects Cell Size and Cell Number Independently

**Figure 6:**
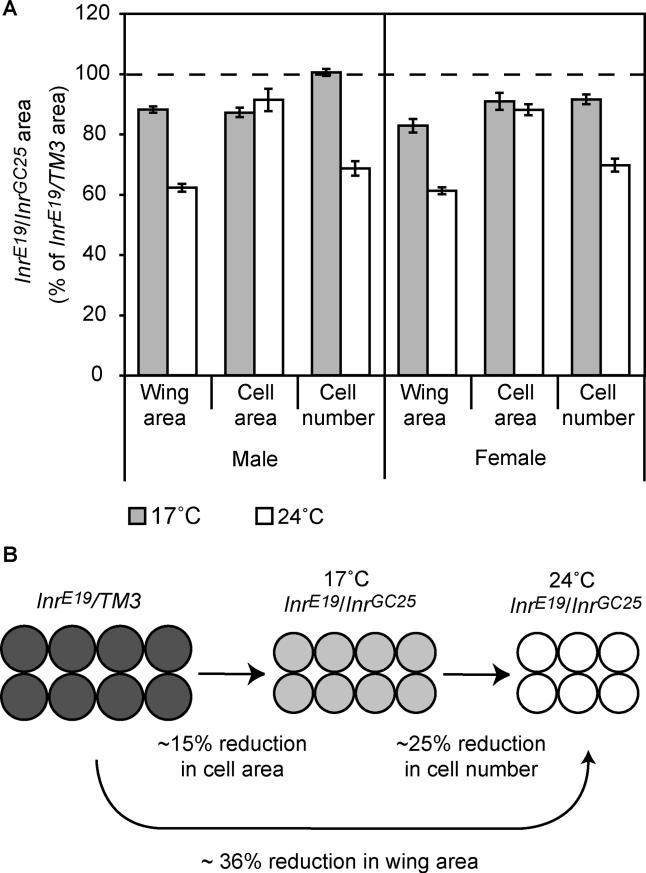
A Reduction in Inr Activity Affects Cell Size and Cell Number Independently (A) At 17 °C, the difference in wing area between *Inr^E19^/Inr^GC25^* and *Inr^E19^/TM3* flies is due to a difference in cell size, whereas at 24 °C the difference is due to an additional difference in cell number. Bars show wing area, cell area, and cell number in *Inr* ^E19^/*Inr* ^GC25^ flies as a percentage of area or number in *Inr^E19^/TM3* flies. (B) At 17 °C the reduced *Inr* activity in *Inr^E19^/Inr^GC25^* flies reduces cell area to approximately 85% the area in *Inr^E19^/TM3* flies, whereas at 24 °C there is no further reduction in cell area, but there is a reduction in cell number to approximately 75% of the number in *Inr^E19^/TM3* flies. Mean wing and cell area, and cell number are given in Table S2.

To investigate the cellular basis for the effect of reduction of Inr activity on size, we compared the size and number of epidermal wing cells in *Inr* ^E19^ */Inr* ^GC25^ flies with *Inr^E19^/TM3* flies, reared at 17 °C and 24 °C. The wing-size difference between *Inr^E19^/Inr^GC25^* and *Inr^E19^/TM3* flies reared at 17 °C is due to smaller cells but not fewer cells in the wings of *Inr^E19^/Inr^GC25^* males (compare relative sizes at 17 °C, Figure 6, Table S2). In *Inr^E19^/Inr^GC25^* females reared at 17 °C, cell size is also smaller than in *Inr^E19^/TM3*females, but there are also slightly fewer cells. In both males and females, the additional difference in wing-size observed in *Inr^E19^/Inr^GC25^* relative to *Inr^E19^/TM3* flies reared at 24 °C is due to fewer, but not smaller, cells: Cell size in *Inr^E19^/Inr^GC25^* wings relative to *Inr^E19^/TM3* wings is the same at both 17 °C and 24 °C (Figure 6, Table S2). Reduction in wing area through suppression of the insulin pathway in *Inr^E19^/Inr^GC25^* flies is therefore predominantly a consequence of smaller cells at 17 °C but fewer cells at 24 °C.

## Discussion

Suppressing the insulin-signaling pathway extends developmental time and reduces final adult size in *Drosophila*. By varying the activity of Inr we have demonstrated that these effects depend on when in development the suppression occurs; Inr suppression affects total developmental time early in development, and body and organ size late in development. The transition from affecting developmental time to affecting body and organ size occurs when the fly reaches the critical size, the point at which development can be completed in the absence of food. The effect of reduced Inr activity on size varies from organ to organ, implicating the insulin pathway in the control of allometry in *Drosophila*. In addition, varying the magnitude of Inr activity has different effects on cell size and cell number.

### Insulin Signaling, Critical Size, and Developmental Delay

We found that a reduction in Inr activity after critical size does not delay development (see Figure 3A). Therefore reducing Inr activity affects total developmental time by delaying the time at which larvae reach critical size. Critical size has been identified as the key stage in insect maturation that determines the point at which, in holometabolous insects, a larva becomes committed to metamorphosis [21]. In Drosophila it is also the minimal viable weight necessary to survive pupation. Consequently, because larvae can complete development without any additional feeding after reaching its critical size, the critical size sets the lower limit of final adult size.

How insects measure critical size is largely unknown. Our results indicate that a reduction in Inr activity delays the point at which *Drosophila* larvae reach critical size. We hypothesize that critical size measurement involves a specific organ or organs, and that it is the slow growth of this organ or organs that delays development in *Inr* mutants. Possible candidates include the imaginal discs and the fat body. Regeneration of damaged imaginal discs delays pupariation in *Drosophila* [22,23], indicating that some or all of the imaginal discs need to grow to a particular size before a larva can pupariate. Complete removal of the discs does not, however, delay pupariation [23]. Another organ must therefore measure critical size and initiate pupariation, with the immature imaginal discs inhibiting pupariation. Slow growth of either the imaginal discs or a “critical-size organ” could delay development in *Inr* mutants. Recent studies suggest this “critical-size organ” could be the fat body, which functions as a nutrient sensor and is involved in the coordination of organismal growth [24]. Suppressing Inr/PI3K signaling in the fat body alone is sufficient to inhibit larval growth and mimics the effects of starvation [18].

### Insulin Signaling and Final Body and Organ Size

A reduction in Inr activity after critical size reduces final adult size and organ size (see Figure 3A and 3C). Inr activity influences adult body size and wing size for different periods of development. As expected, adult body size is influenced by Inr activity only between critical size and pupariation, after which the larva does not feed and becomes a “closed system,” neither gaining nor losing mass. However, insulin signaling continues to influence the final size of adult organs well into the pupal stage. *Inr^E19^/Inr^GC25^* flies switched from 17 °C to 24 °C at pupariation have the same mass as *Inr^E19^/Inr^GC25^* flies maintained at 17 °C, but have reduced wings. At the same time, the temperature-shifted flies have a much higher free-sugar concentration as adults. These two findings appear to be linked. Considerable cell proliferation in the imaginal discs occurs after the larva has stopped feeding [11,12,25], and this proliferation relies entirely on stored nutrients as an energy source. Nutrient storage occurs predominantly in the fat body cells, which accumulate reserves of proteins, lipids, and glycogen (the major carbohydrate storage compound) during the third larval instar [18]. Both starvation and suppression of the insulin pathway cause these nutrients to be mobilized for use by growing cells [18]. The finding that *Inr^E19^/Inr^GC25^* mutants reared at 24 °C have elevated free-sugar levels, but do not have elevated glycogen levels, indicates that they are able to mobilize their carbohydrate reserves, but that the free-sugars are not taken up by growing organs, and remain in the haemolymph.

### Insulin Signaling and Cell Size and Cell Number

Any mechanism that influences organ size does so by changing cell size, cell number, or both. Although mutations of *Inr*, *PI3K*, and *chico* reduce both cell size and cell number [4,5,13,26], downstream components of the insulin-signaling pathway can affect cell size and cell number independently. The RPS6-p70-protein kinase (S6K) branch of the pathway appears to influence cell size but not cell number [27], whereas the dFOXO branch appears to influence cell number but not cell size [15,16]. Because these signaling branches diverge downstream of the insulin receptor, it has not been clear how insulin signaling could affect cell size and number differentially. These changes are ultimately a consequence of changes in the relative rates of cell growth and division [28]. For example, the reduction in cell size, but not cell number, in *S6K* mutants implies a reduction in the rate of cell growth but not of cell division. Conversely, the reduction in cell number, but not cell size, when *dFOXO* is over-expressed implies that the rates of cell growth and division are reduced equally. Finally, a reduction in both cell size and cell number will result if both the rates of cell growth and division are reduced, if the former is reduced to a greater extent than the latter.

Our results support the hypothesis that insulin signaling differentially affects cell size and cell number via different levels of insulin receptor activity. Slightly reduced levels of activity, as in *Inr^E19^/Inr^GC25^* flies raised at 17 °C, reduce cell size, possibly through a reduction in the rate of cell growth but not of cell division. Further reductions in the levels of activity, as in *Inr^E19^/Inr^GC25^* flies raised at 24 °C, reduces cell number only, possibly through a subsequent balanced reduction in both the rate of cell growth and cell division. These data are consistent with a result from Bohni et al. [5]. They showed that the wings of *chico^2^* homozygotes are smaller due to a reduction of both cell size (17%) and cell number (27%). However, a further reduction in insulin signaling through the removal of a single copy of *Inr* enhances the small-size phenotype exclusively through a reduction in cell number but not cell size. Although we cannot exclude the possibility that the differences in cell size between *Inr^E19^/Inr^GC25^* and *Inr^E19^/TM3* flies are a consequence of genetic background unrelated to differences in Inr activity, the reduction in cell number alone is clearly due to reduction in insulin signaling (see Figure 5).

### Insulin Signaling and Allometry

Variation in insulin signaling appears to affect the allometric relationship between organs. For example, at 17 °C, male *Inr^E19^/Inr^GC25^* flies have body size, wings, maxillary palps, and genital arches approximately 85% of wild-type. Increasing the rearing temperature of *Inr^E19^/Inr^GC25^* flies from 17 °C to 24 °C, however, causes a further reduction in wing, maxillary palp, and overall body size but does not affect the size of the genital arches (Figure 5). Consequently, flies reared at 24 °C have larger genitals relative to their body and wings compared to flies reared at 17 °C. The apparently restricted response of the genitals to changes in insulin signaling is not a consequence of the particular alleles used in this study: *chico*-mutant clones also have much less of an effect on size when they are in the genital arches than when they are in the maxillary palps.

The mechanism by which different organs respond differently to changes in insulin signaling is unclear. Organs may vary in their expression of the insulin receptor gene or may limit the activity of certain downstream components of the insulin signaling pathway. In 17 °C *Inr^E19^/Inr^GC25^* males, the genital arches, wings, and maxillary palps are all reduced by approximately the same amount relative to 17 °C *Inr^E19^/TM3*males. In the wing, this reduction in area is a consequence of a reduction in cell size. A further decrease in Inr activity (through an increase in rearing temperature to 24 °C) reduces wing area through a reduction in cell number alone. If the genital arches are like the wing, then their response to insulin signaling may be restricted to changes in cell size and not cell number. The cells of the genital arches may therefore be deficient in components of the insulin-signaling pathway that regulate cell number but not components that regulate cell size.

### A Model of the Insulin-Signaling Regulation of Growth and Development

**Figure 7:**
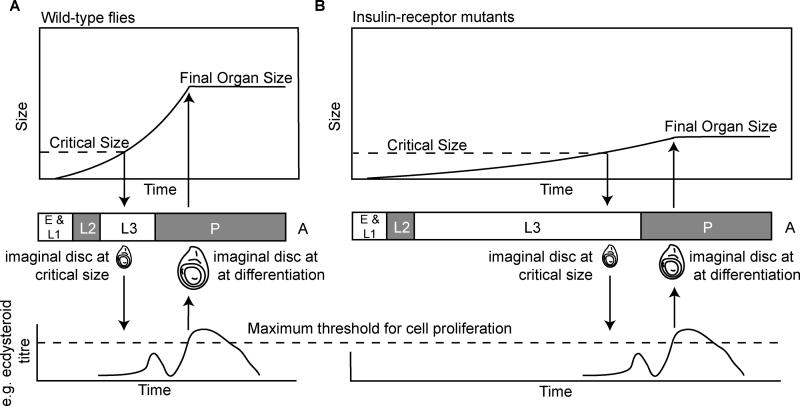
A Model of the Insulin-Signaling Regulation of Growth and Development (A) Under normal conditions, imaginal discs grow to a critical size, which initiates an increase in the ecdysteroid titer. When ecdysteroid levels rise above a maximum threshold, the discs cease cell proliferation and undergo differentiation, fixing their final size. A, adult; E, embryo; L1–L3, first to third larval instar; P, pupa. (B) In *Inr* mutants, growth of imaginal discs to critical size is slowed, retarding development. When critical size is reached, the ecdysteroid titer again increases, rising above the maximum threshold for cell proliferation in the imaginal discs. Temporal changes in the ecdysteroid titer are unaffected by insulin signaling. Because the rate of cell proliferation is slowed, the imaginal discs are smaller when they begin to differentiate, reducing final organ size. Different discs have different thresholds of sensitivity to ecdysteroid and so cease cell proliferation at different times. Hormones other than ecdysteroids may also be involved.

The insulin-signaling pathway appears to play a different role after critical size than before. Similarly, a temperature-sensitive lethal mutation, *l(1)ts-1126,* which reduces the rate of cell proliferation in *Drosophila,* delays pupariation when larvae are moved to a restrictive temperature before the third instar, but reduces adult size when larvae are moved to a restrictive temperature late in the third instar [29]. The two effects of reduced Inr activity may therefore result from the same process: a reduction in the rate of cell growth and proliferation. We have developed a model of the insulin-signaling regulation of growth and development in *Drosophila* (Figure 7). (A similar model has recently been developed by Davidowitz and Nijhout [30] to explain variation in body size in response to temperature in the tobacco hornworm, *Manduca sexta.*) A reduction in Inr activity prior to critical size slows cell growth and proliferation and delays the time at which the larvae reaches critical size. Critical size is not substantially influenced by nutritional conditions [8] or insulin signaling in *Drosophila,* although this does not seem to be the case for all insects, for example, *M. sexta* [21]. Once critical size is reached, the time to pupariation and adult eclosion is fixed, as are the remaining periods of growth prior to adult differentiation of individual imaginal discs. The duration of these intervals are uninfluenced by nutritional conditions or insulin signaling. A reduction in Inr activity during these periods also slows cell growth and proliferation, but now reduces the amount of growth attained before differentiation, resulting in smaller organs and a smaller fly. Because different structures grow for different periods, they are sensitive to Inr activity at different times. For example, wing size begins to be insensitive to changes in Inr activity at approximately the same time as cell proliferation ceases, around 25% into the pupal stage. Adult body mass becomes insensitive to changes in Inr activity just before pupariation, when the larvae stops feeding and final body size is fixed.

A key component of this model is that after critical size, the remaining periods of growth of individual imaginal discs and of the body as a whole are fixed and uninfluenced by insulin signaling. Our data show that the length of the period between critical size and pupariation, the remaining period of growth of the body as a whole, is not substantially affected by a reduction in Inr activity. In *Drosophila* and other insects, this interval is controlled by endocrine events. In *Drosophila,* a small peak in the ecdysteroid titer coincides with attainment of critical size [31], followed by a second peak 12 h later, just before the larvae leave the food [32]. In *M.* *sexta* this second peak acts directly on the nervous system to initiate wandering behavior [33], and the same is likely true for *Drosophila* [34]. The period in which Inr activity can influence final body size is therefore terminated by hormones. Importantly, hormones other than insulin also control the period of cell proliferation in the imaginal discs. For example, in *M.* *sexta*, ecdysteroids govern the phases of eye development during metamorphosis [35,36]. When the ecdysteroid titer rises above a minimum threshold just before pupariation, it stimulates a wave of cell proliferation to pass across the eye primordium. This proliferation is sustained until the ecdysteroid titer rises above a maximum level in the middle of pupal development, whereupon cell proliferation stops and maturation of the ommatidia begin. These and similar data in other insects [37–39], including *Drosophila* [40–43], suggest that cell proliferation in imaginal discs may be temporally regulated by thresholds of sensitivities to fluctuating levels of ecdysteroids and juvenile hormone (JH) [44]. Different organs have different thresholds of sensitivity and hence grow for different periods of time. Insulin signaling may therefore control body and organ size by regulating the amount of growth attained during these periods of cell proliferation.

This model requires that changes in ecdysteroid and JH levels are unaffected by the insulin-signaling pathway. It is known that adult *Inr* and *chico* mutant flies have reduced levels of JH and impaired ovarian ecdysone synthesis [45,46]. However, the same hormone fluctuations that putatively control the period of cell proliferation also initiate pupariation [34] and, because the timing of pupariation is unaffected by Inr activity, it seems likely that the temporal dynamics of the hormonal cascade in the larvae are also unaffected by Inr activity. We predict, therefore, that the insulin-signaling pathway regulates cell proliferation in imaginal discs but that the duration of proliferative phases are controlled by other endocrine cues, such as JH and ecdysteroids, that are themselves unaffected by the insulin-signaling pathway.

This model demonstrates how the pleiotropic effects of insulin signaling on developmental time and final body and organ size can be separated, and may be available for independent evolutionary modification. For example, changes in the relative size of an organ may occur by organ-specific modifications in its growth response to insulin signaling, through organ-specific changes in the expression of *Inr*, adjustments in Inr activity, or adjustments in the expression and activity of downstream components of the insulin-signaling pathway. Alternatively, changes in the period of an organ's growth, through alterations in its sensitivity to other endocrine cues, may have a similar effect [44]. In metazoans in general, each organ has a unique timetable for cellular events in tissue development. Our model, and the data upon which it is based, indicate that in order to understand the effects of insulin signaling on adult phenotype it is necessary to understand how temporal changes in insulin signaling interact with this timetable.

## Materials and Methods

### Mutant stocks

*Inr^GC25^* is a chromosomal inversion with a breakpoint upstream of the *Inr*. *Inr^E19^* is an uncharacterized mutation induced by ethyl methanosulfonate. Both were described in Chen et al. [4] and obtained from the Bloomington Stock Center. The *chico* ^1^ allele is a P-element insertion allele whose phenotype is similar to the null *chico* ^2^ allele [5] and was kindly provided by Ernst Hafen. The *tub-GFP-PH* flies were kindly supplied by Bruce Edgar. The flies were maintained as *chico^1^/CyO* [5], *Inr^E19^_,_ and Inr^GC25^* [4] balanced over TM6B-Tb, TM3, TM3-pAct-GFP, and tub-GFP-PH; *Inr* ^E19^/TM3 [18] at 17 °C on standard yeast cornmeal agar medium.

### Immunolocalization

We crossed *Inr^E19^/TM6-Tb* with *Inr^GC25^/TM6-Tb* flies and reared them at either 17 °C or 24 °C. When the larvae reached third instar, we genotyped them as either insulin receptor mutants (*Inr^E19^* */Inr^GC25^*) or wild-type (*Inr^E19^* or *Inr^GC25^*/*TM6B-Tb*). The fat bodies were dissected out in PBS, fixed in 4% paraformaldehyde for 10 min, and stored in absolute methanol at −20 °C. They were permeabilized with PBT (0.3% Triton X in PBS) for 30 min, washed in BBT (0.3% bovine serum albumin in PBT) for 30 min, then blocked in NGS/BBT (3% normal goat serum in BBT) for 30 min. They were stained with anti-dFOXO 2095 [16] (kindly provided by O. Puig) (1:1,000 in PBT) and fluorescein anti-rabbit (1:500 in PBT) (Vector Labs, Burlingame, California, United States). DNA was stained with propidium iodide (1:1,000 in PBS with 1 μg of RNase A). We mounted the fat bodies in Vectashield (Vector Labs) for observation under a confocal microscope (Perkin Elmer UltraVIEW RS3; PerkinElmer Life and Analytical Sciences, Boston, Massachusetts, United States).

### GPH localization

We crossed *tub-GFP-PH;Inr^E19^/TM6B-Tb* with *Inr* ^GC25^/*TM6B-Tb* flies and reared them at 15 °C. When the larvae reached second instar, they were removed from their food, washed, and genotyped as insulin receptor mutants (*tub-GFP-PH;Inr^E19^/Inr^GC25^*) or wild-type (*tub-GFP-PH;Inr^E19^* or *Inr^GC25^/TM6B-Tb*). They were then transferred to fresh food and maintained at 15 °C or 25 °C for 24 h. We then dissected the larvae in 100% methanol kept at 15 °C or 25 °C depending on the temperature treatment of the larvae, and stored their fat bodies without additional fixing in 100% methanol at −20 °C. We quickly washed the fat bodies in 50% methanol in PBS, and then 100% PBS before mounting them in Vectashield with Hoechst 33342 (2:1,000) to stain DNA. We observed the fat bodies under a confocal microscope (Zeiss LSM 510; Zeiss, Gena, Germany).

### Temperature shift

We reared flies at a number of temperature regimes. They were either maintained at 17 °C, 18 °C, 24 °C, or 25 °C or switched from 17 °C to 24 °C on day 1 to day 27 of development. We set up four bottles for each temperature regime, each containing standard yeast cornmeal agar medium. Two were the product of male *Inr^E19^/TM6B-Tb* and female *Inr^GC25^/TM3* and two were the product of female *Inr^E19^/TM6B-Tb* and male *Inr^GC25^/TM3*. Each bottle contained between 100 and 200 eggs laid over a 4-h period. Temperature shifts were performed according to the median age of flies in each bottle. Larvae were reared on standard yeast cornmeal agar medium. Total developmental time was not significantly different between *Inr^E19^/Inr^GC25^* flies from the two different crosses, so the data from all bottles maintained under the same temperature regime were pooled (data not shown).

### Measurements and data handling

Bottles were inspected daily at 4:00 PM and any flies that had eclosed in the previous 24 h were collected and preserved in 80% ethanol. Flies were then genotyped and the eclosion times to the nearest day of each *Inr^E19^/Inr^GC25^* and *Inr^E19^/TM3* control fly was recorded. We used *Inr^E19^/TM3* rather than *Inr^GC25^/TM6B-Tb* flies as controls because *TM6-Tb* has *Tubby* as a marker, which affects developmental timing and adult shape [47]. Ten to 15 *Inr^E19^/Inr^GC25^* and *Inr^E19^/TM3* female wings from each temperature regime were dissected and mounted in lactic acid:ethanol (4:5). Ten *Inr^E19^/Inr^GC25^* and *Inr^E19^/TM3* males reared at 24 °C and 15 *Inr^E19^/Inr^GC25^* and *Inr^E19^/TM3* males reared at 17 °C were also dissected and their wings, maxillary palps, and genital arches mounted in either lactic acid:ethanol (wings) or Hoyer's Solution (maxillary palps and genital arches). Digital images of the wings, maxillary palps, and genital arches were captured and measured using IPLab 3.9 (Scanylitics, Fairfax, Virginia, United States). Wing cell size was estimated using the number of trichomes in a 0.01 mm^2^ square between the veins IV and V of the dorsal wing blade. An index of the total number of wing cells was estimated by multiplying the number of trichomes in the area by the total wing size and dividing by 0.01. Dry masses of individual male flies were measured with an analytical balance (Mettler Toledo AX26 DeltaRange; Mettler-Toledo, Columbus, Ohio, United States).

The total developmental time for each fly was converted into degree-days (DDs) to control for the effects of temperature on growth rates. DD is a measure of the heat accumulation above a minimal temperature (T_o_), and is calculated as:



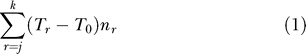



where *T*_r_ is the experimental temperature, and *n*_r_ is the number of days maintained at *T*_r_, summed across all experimental temperatures *j* to *k*. In this case, we used only two experimental temperatures, 17 °C and 24 °C. We calculated the value of *T*_0_ such that the developmental time of control *Inr^E19^/TM3* flies was constant, irrespective of temperature regime. To do this we regressed total developmental time in DDs of *Inr^E19^/TM3* flies against the time of their temperature shift from 17 °C to 24 °C, using different values of *T*_0_. The value of *T*_0_ that minimized the slope of this regression line was found to be 9.978 °C. When *T*_0_ is 9.978 °C, the average developmental time of *Inr^E19^/TM3* flies is 156.04 ± 0.338 DD. We then converted the total developmental time for each *Inr^E19^/Inr^GC25^* fly into DDs using *T*_0_ = 9.978, and expressed it as the average percentage of developmental time of *Inr^E19^/TM3* flies (156.04 DD), along with the standard error of the percentages.

Rearing temperature also affects body, organ, and cell size and cell number [19]. Consequently, all these measurements in *Inr^E19^/Inr^GC25^* flies were expressed as a percentage of the value in *Inr^E19^/TM3* flies maintained under the same temperature regime. Wing area of *Inr^E19^/Inr^GC25^* females and dry mass of *Inr^E19^/Inr^GC25^* males switched from 17 °C to 24 °C increasingly late in development, was expressed as the percentage of wing area and dry mass of *Inr^E19^/TM3* flies switched from 17 °C to 24 °C at the same percent of total development at 17 °C. See Figure S1 for details.

### Clonal analysis

We crossed males carrying the alleles *chico^1^*/*CyO* with females carrying the alleles *f^36a^*;*M(2)Z f+37C/CyO* . Larvae were subjected to X-rays (1,000 rad) at 24–72 h after egg-laying. Genitals and maxillary palps of male offspring without balancer chromosomes were studied for *f^36a^* bristles.

### Developmental staging of *Inr^E19^/Inr^GC25^* and *Inr^InrE19^/TM3* at 17 °C and 24 °C

We crossed *Inr^E19^/TM3-pAct-GFP* flies with *Inr^GC25^/TM3-pAct-GFP* flies and collected the eggs over a period of 4 h. After 24 h, we selected *Inr^E19^/Inr^GC25^* and *Inr^E19^* or *Inr^GC25^* */TM3-pAct-GFP* eggs using a fluorescence microscope to detect GFP activity. The eggs were transferred to 12 mm ø Petri dishes containing standard yeast cornmeal agar medium. Each dish contained 20 eggs of only one genotype and was maintained at either 17 °C or 24 °C. We prepared three dishes: *Inr^E19^/Inr^GC25^* maintained at 17 °C; *Inr^E19^/Inr^GC25^* maintained at 24 °C; and *Inr^E19^* or *Inr^GC25^* */TM3-pAct-GFP* maintained at 17 °C. From day 2, ten individuals were randomly selected from each Petri dish and their developmental stage was recorded using mouthpart development, before being returned to the Petri dish.

### Body chemistry assays

White prepupae of *Inr^E19^/Inr^GC25^* and *Inr^E19^/TM3* flies raised at 17 °C were transferred to moistened Kimwipes (Kimberly-Clark, Neenah, Wisconsin, United States) in Petri dishes. Prepupae of each genotype were split between further development at 17 °C and 24 °C. We collected adults within 8 h of eclosion, after their cuticle had hardened, and immediately froze them in liquid nitrogen for later analysis. Wet and dry masses of individual flies were measured with an analytical balance (Mettler Toledo AX26 DeltaRange). All metabolic assays were performed on individual dried males (*n* = 10 for each assay). Glycogen and sugar content were measured using a protocol of van Handel [48]. Lipid content was quantified using another protocol of van Handel [49]. To determine protein concentration, flies were homogenized in 100 μl 0.1 M Na_2_HPO_4_. Ten μl of each sample was combined with the Bio-Rad protein assay dye reagent (Bio-Rad Laboratories, Hercules, California, United States) following the manufacturer's instructions and spectrophotometrically assayed at 595 nm.

## Supporting Information

Figure S1 Fitted Values for Wing Aarea of *Inr^E19^/TM3* Control Flies Wing area of *Inr^E19^/Inr^GC25^* flies in Figure 2 is expressed as percentage of wing area of *Inr^E19^/TM3* flies kept under the same temperature regime, using the fitted values shown on the plot. Temperature affects wing area differently before and after critical size. Consequently, fitted values were calculated by regressing wing area of *Inr^E19^/TM3* flies against transfer age before critical size (35% development) and after critical size. A similar method was used to determine the dry mass of *Inr^E19^/TM3* males, except a single regression analysis was used to calculate the fitted values.
(59 KB PDF).

Table S1 Wing, Genital Arch, and Maxillary Palp Area of *Inr^E19^/Inr^GC25^* and *Inr^E19^/TM3* Males Reared at 17 °C and 24 °C (37 KB DOC).

Table S2 Total Area, Cell Area, and Cell Number of the Wings of *Inr^E19^/Inr^GC25^* and *Inr^E19^/TM3* Flies Reared at 17 °C and 24 °C (45 KB DOC).

### Accession Numbers

The FlyBase (http://flybase.bio.indiana.edu/search/) accession numbers for the genes and gene products discussed in this paper are Chico (Fbgn0024248), chico1 (FBal0031303), dFOXO (FBgn0038197), Inr (FBgn0013984), InrE19 (FBal0094021), InrGC25 (FBal0010755), PI3K (Fbgn0015279), and S6K (FBgn0015806). The National Center for Biotechnology Information (NCBI) (http://www.ncbi.nlm.nih.gov/) accession number for IGF1 receptor is NM_010513.

## Abbreviations

DD: degree day
JH: juvenile hormone
PI3K: phosophoinositide 3-kinase
TSP: temperature sensitive period
