# Supplementary material for: The Temporal Requirements for Insulin Signaling During Development in Drosophila
Source: PLoS Biol. 2005 Aug 16;3(9):e289. doi: 10.1371/journal.pbio.0030289 (PMC1184592; doi:10.1371/journal.pbio.0030289)
Supplement: Supplementary file 1 [file pbio.0030289.sg001.pdf]

Figure S1

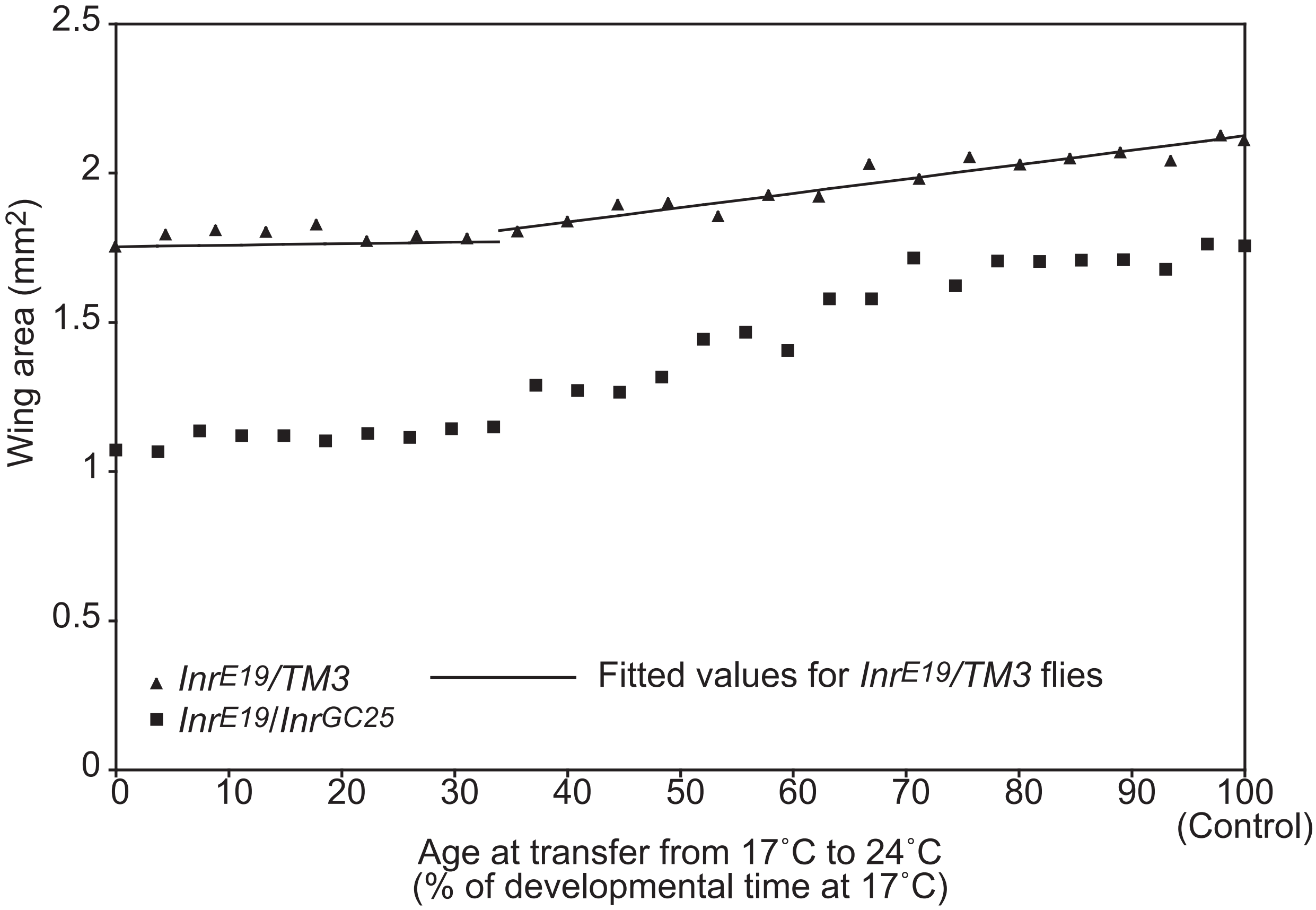

Wing area (mm^2)
2.5
2
1.5
1
0.5
0
InrE19/TM3
InrE19/InrGC25
Fitted values for InrE19/TM3 flies
0
10
20
30
40
50
60
70
80
90
100
(Control)
Age at transfer from 17˚C to 24˚C
(% of developmental time at 17˚C)
