# Supplementary material for: The Temporal Requirements for Insulin Signaling During Development in Drosophila
Source: PLoS Biol. 2005 Aug 16;3(9):e289. doi: 10.1371/journal.pbio.0030289 (PMC1184592; doi:10.1371/journal.pbio.0030289)
Supplement: Supplementary file 2 [file pbio.0030289.st001.doc]

Supplementary Table S1: Wing, genital arch and maxillary palp area of *Inr^E19^/Inr^GC25^* and *Inr^E19^/TM3* males reared at 17°C and 24°C.

|  | Rearing ­­Temperature | Genotype | | F-test | |
| --- | --- | --- | --- | --- | --- |
| Organ area |  | *Inr^E19^/TM3* | *Inr^E19^/Inr^GC25^* | *(genotype)^a^* | (genotype*temperature)^b^ |
| Wing (mm^2^) | 17°C | 1.74 ± 0.0110 | 1.53 ± 0.0130 | F_1,46_ = 753.6***** | F_1,46_ = 135.4***** |
|  | 24°C | 1.37 ± 0.0130 | 0.85 ± 0.0172 |  |  |
|  |  |  |  |  |  |
| Genital arch (μm^2^) | 17°C | 4290 ± 83.7 | 3610 ± 82.8 | F_1,46_ = 61.7***** | F_1,46_ = 0.3 |
|  | 24°C | 3690 ± 79.8 | 2920 ± 113 |  |  |
|  |  |  |  |  |  |
| Maxillary palp (μm^2^) | 17°C | 11400 ± 236 | 9910 ± 120 | F_1,46_ = 208.1***** | F_1,46_ = 43.5***** |
|  | 24°C | 9990 ± 172 | 6080 ± 139 |  |  |

^a^ Tests whether organ area differs between *Inr*^E19^/*Inr*^GC25^ and *Inr*^E19^/TM3 independent of temperature.

^b^ Test whether the effect of temperature on organ area differs between *Inr*^E19^/*Inr*^GC25^ and *Inr*^E19^/TM3 flies, that is, whether a reduction in Inr activity in temperature sensitive *Inr*^E19^/*Inr*^GC25^ flies influences organ area.

* *p* < 0.05 ** *p* < 0.01 *** *p* < 0.0001
