# Supplementary material for: The Temporal Requirements for Insulin Signaling During Development in Drosophila
Source: PLoS Biol. 2005 Aug 16;3(9):e289. doi: 10.1371/journal.pbio.0030289 (PMC1184592; doi:10.1371/journal.pbio.0030289)
Supplement: Supplementary file 3 [file pbio.0030289.st002.doc]

Supplementary Table S2: Total area, cell area and cell number of the wings of *Inr^E19^/Inr^GC25^* and *Inr^E19^/TM3* flies reared at 17°C and 24°C

|  | Rearing Temperature | Genotype | | F-test | |
| --- | --- | --- | --- | --- | --- |
|  |  | *Inr^E19^/TM3* | *Inr^E19^/Inr^GC25^* | *(genotype)^a^* | (genotype*temperature) |
| Male |  |  |  |  |  |
| Wing area (mm^2^) | 17°C | 1.74 ± 0.0106 | 1.53 ± 0.0181 | F_1,44_ = 509.2 ***** | F_1,44_ = 92.5***** |
|  | 24°C | 1.36 ± 0.0173 | 0.85 ± 0.0180 |  |  |
|  |  |  |  |  |  |
| Cell area (μm^2^) | 17°C | 196 ± 4.86 | 171 ± 3.07 | F_1,44_ = 14.95**** | F_1,44_ = 2.28 |
|  | 24°C | 149 ± 3.30 | 138 ± 6.51 |  |  |
|  |  |  |  |  |  |
| Cell number index^c^ | 17°C | 8963± 224 | 9002 ± 106 | F_1,44_ = 47.90***** | F_1,44_ = 50.6***** |
|  | 24°C | 9134 ± 211 | 6209 ± 257 |  |  |
| Female |  |  |  |  |  |
| Wing area (mm^2^) | 17°C | 2.10 ± 0.0435 | 1.74 ± 0.0110 | F_1,36_ = 274.8***** | F_1,36_ = 25.47***** |
|  | 24°C | 1.75 ± 0.0384 | 1.07 ± 0.0199 |  |  |
|  |  |  |  |  |  |
| Cell area (μm^2^) | 17°C | 211 ± 5.05 | 192 ± 5.00 | F_1,36_ = 19.22***** | F_1,36_ = 0.0211 |
|  | 24°C | 174 ± 4.84 | 153 ± 3.12 |  |  |
|  |  |  |  |  |  |
| Cell number index^c^ | 17°C | 9999 ± 232 | 9146 ± 270 | F_1,36_ = 75.71***** | F_1,36_ = 24.20***** |
|  | 24°C | 10115 ± 167 | 7045 ± 220 |  |  |

^a^ Tests whether wing area cell area or cell number differ between *Inr*^E19^/*Inr*^GC25^ and *Inr*^E19^/TM3 independent of temperature.

^b^ Test whether the effect of temperature on area or number differs between *Inr*^E19^/*Inr*^GC25^ and *Inr*^E19^/TM3 flies, that is, whether a reduction in Inr activity in temperature sensitive *Inr*^E19^/*Inr*^GC25^ flies influences area or number.

^c^ Index is derived from measure of wing area and cell area. * *p* < 0.05 ** *p* < 0.01 *** *p* < 0.0001
